# PHLDA1 Suppresses TLR4-Triggered Proinflammatory Cytokine Production by Interaction With Tollip

**DOI:** 10.3389/fimmu.2022.731500

**Published:** 2022-02-14

**Authors:** Hui Peng, Juping Wang, Xuhong Song, Jiangni Huang, Haoming Hua, Fanlu Wang, Ziyun Xu, Jing Ma, Jie Gao, Jing Zhao, Anna Nong, Dongyang Huang, Bin Liang

**Affiliations:** ^1^ Department of Cell Biology and Genetics, Key Laboratory of Molecular Biology in High Cancer Incidence Coastal Chao Shan Area of Guang Dong Higher Education Institutes, Shantou University Medical College, Shantou, China; ^2^ Department of Clinical Laboratory, Affiliated Hospital of Youjiang Medical University for Nationalities, Baise, China; ^3^ Department of Pathophysiology, School of Basic Medical Sciences, Youjiang Medical University for Nationalities, Baise, China

**Keywords:** PHLDA1, TLR4, suppress, proinflammatory cytokine, Tollip

## Abstract

Pleckstrin homology-like domain, family A, member 1 (PHLDA1) has been reported to be expressed in many mammalian tissues and cells. However, the functions and exact mechanisms of PHLDA1 remain unclear. In this study, we found that PHLDA1 expression was significantly altered in macrophages after exposure to lipopolysaccharide (LPS) *in vitro*, suggesting that PHLDA1 may be involved in the regulation of TLR4 signaling pathway activated by LPS. PHLDA1 attenuated the production of LPS-stimulated proinflammatory cytokines (TNF-α, IL-6, and IL-1β). Further research showed that the phosphorylation levels of some important signal molecules in TLR4/MyD88-mediated MAPK and NF-κB signaling pathways were reduced by PHLDA1, which in turn impaired the transcription factors NF-κB and AP1 nuclear translocation and their responsive element activities. Furthermore, we found that PHLDA1 repressed LPS-induced proinflammatory cytokine production *via* binding to Tollip which restrained TLR4 signaling pathway. A mouse model of endotoxemia was established to confirm the above similar results. In brief, our findings demonstrate that PHLDA1 is a negative regulator of LPS-induced proinflammatory cytokine production by Tollip, suggesting that PHLDA1 plays an anti-inflammatory role through inhibiting the TLR4/MyD88 signaling pathway with the help of Tollip. PHLDA1 may be a novel therapeutic target in treating endotoxemia.

## Introduction

Pleckstrin homology-like domain, family A, member 1 (PHLDA1), which is also called T-cell death-associated gene 51 (TDAG51), was first found to induce apoptosis through cross-linking T-cell receptor (TCR) signaling pathway to Fas expression (1). The PHLDA1 family consists of three members, including PHLDA1, PHLDA2, and PHLDA3. PHLDA1, which contains pleckstrin homology−like domain, polyglutamine tract, proline−glutamine tract, and proline−histidine−rich tract (2, 3), has three splice variants (PHLDA1-201, 202, and 203) and encodes a protein with 401 amino acids (45 kDa) or 260 amino acids (29.7 kDa) in length. Numerous studies have confirmed PHLDA1 expression at the protein and mRNA levels in many mammalian tissues, including the brain, endocrine tissues, and proximal digestive tract. PHLDA1 was also found to express in many types of cancer, such as brain, liver, bladder, and lung cancer (2, 4, 5). Studies have suggested that PHLDA1 is involved in many biological processes, such as cell proliferation, cell differentiation, cell death, cancer metastasis, epithelial–mesenchymal transition, and cancer stem cell properties (6–9). Although PHLDA1 has gotten increasing attention over the past 20 years, its role in endotoxemia remains to be elucidated.

The innate immune response eliminates invading microorganisms by inducing proinflammatory molecules (cytokines, chemokines, antibiotics, etc.) (10, 11). Toll-like receptors (TLRs) are an important component of innate immune response. The discovery of TLRs promoted the development of innate immunity. The first human homolog of the Drosophila Toll protein, now known as TLR4, was discovered by Janeway and colleagues in 1997 (12, 13). TLR4 is widely distributed in a variety of tissues such as the liver, colon, kidney, and spleen. TLR4 is also expressed in various cells such as Kupffer cells, monocytes, macrophages, neutrophils, Hofbauer cells, and cancer cells (14–16). Upon LPS stimulation, TLR4 forms the MD2/TLR4/CD14 complex (17–20) and causes the release of proinflammatory mediators by two classical signaling pathways, namely, MyD88-dependent signaling pathway and MyD88-independent signaling pathway, and then induces an immune response eventually (21–25).

In this study, the kinetics of PHLDA1 expression was first confirmed upon stimulation with LPS *in vitro*, which prompted us to focus on the role of PHLDA1 in the modulation of the TLR4 signaling pathway. We further found that PHLDA1 markedly reduced the phosphorylation levels of some signal molecules (ERK, JNK, p38, IKKα/β, IκBα, and NF-κB subunit p65) in TLR4/MyD88-mediated MAPK and NF-κB signaling pathways, which in turn decreased the production of proinflammatory cytokines (TNF-α, IL-6, and IL-1β). Finally, we verified that LPS induced the increased interaction between PHLDA1 with Tollip. In short, our study reveals a new function of PHLDA1 as a negative regulator of LPS-induced proinflammatory cytokine production.

## Materials and Methods

### Reagents, Antibodies, Plasmids, and Mice

LPS-EK (LPS from *Escherichia coli* K12) was purchased from InvivoGen (San Diego, CA, USA). Transfection reagents, including Lipofectamine 2000 and Lipofectamine RNAiMAX, were ordered from Invitrogen (Camarillo, CA, USA). EZ Cell Transfection Reagent II was ordered from Life-iLab Biotech (Shanghai, China). 4,6-Diamino-2-phenyindole (DAPI) was ordered from Beyotime Biotechnology (Shanghai, China). Mouse macrophage colony-stimulating factor (M-CSF) was ordered from PeproTech (East Windsor, NJ, USA). Protein G PLUS-Agarose was ordered from Santa Cruz Biotechnology Inc. (Santa Cruz, CA, USA), and 10× RIPA lysis buffer was ordered from Merck Millipore (Bedford, MA, USA). NF-κB and Renilla luciferase reporter plasmids were gifts from Professor Y. Eugene Chin (Institute of Biology and Medical Sciences, Soochow University Medical College). AP1 luciferase reporter plasmid was obtained from Beyotime Biotechnology (Shanghai, China). The PHLDA1 plasmid was obtained from Genechem (Shanghai, China). The primary and second antibodies are shown in **Supplementary Table 1**. BALB/c mice and C57BL/6J (4–6 weeks old) were purchased from Vital River Laboratory Animal Technology (Beijing, China). All animal experiments were performed in accordance with the guidelines of Youjiang Medical University for Nationalities.

### Cell Culture

RAW264.7, 293T, and L-929 cells were cultured in Dulbecco’s modified Eagle’s medium (DMEM, Invitrogen, Carlsbad, CA, USA) with 10% (v/v) fetal bovine serum (FBS, Gemini Bio-Products, Woodland, CA, USA), 1% streptomycin–penicillin mixtures (Beyotime Biotechnology, Shanghai, China), and 0.03% L-glutamine at 37°C in a humidified atmosphere with 5% CO_2_. Bone marrow-derived macrophages (BMDM) were isolated from the femurs of C57BL/6J mice and cultured in DMEM with 10% (v/v) FBS and M-CSF (10 ng/ml). A total of 4 × 10^4^ cells were seeded into 96-well plates for luciferase reporter activity assay, 2 × 10^5^ cells were cultured in 24-well plates for enzyme-linked immunosorbent assay (ELISA), and 8 × 10^5^ cells were cultured in a 3.5-cm dish for Western blot analysis.

### RNA Interference and Plasmid Transfection

Small interfering RNA (siRNA) for PHLDA1 and Tollip were designed and synthesized by GenePharma (Shanghai, China). The above siRNA fragments were transfected into RAW264.7, BMDM, or L-929 cells using jetPEI^®^-Macrophage transfection reagent (Polyplus Transfection, Illkirch, France) or Lipofectamine RNAiMAX according to the manuals of the manufacturer. The above cells were further analyzed at 72 h after transfection. PHLDA1 and Tollip siRNA sequences are listed in **Supplementary Table 2**. Plasmids were transfected into RAW264.7, BMDM, L-929, or 293T cells using Lipofectamine 2000, jetPEI^®^-Macrophage transfection reagent, or EZ Cell Transfection Reagent II according to the manuals of the manufacturer. The above cells were further analyzed at 48 h after transfection.

### Measurement of Proinflammatory Cytokines

RAW264.7 cells or BMDM were seeded into 24-well plates and transfected as described above after incubation overnight. After 48 h, the above cells were stimulated with LPS for the different time periods. IL-6, TNF-α, and IL-1β in the supernatants were measured with ELISA kits (Elabscience Biotechnology, Wuhan, Hubei, China) according to the instructions of the manufacturer.

### Dual-Luciferase Reporter Assay

293T cells were seeded into 96-well plates and transfected as described above after incubation overnight. After 24 h, the above cells were stimulated with LPS for 20 h. After the above cells were lysed with 1× passive lysis buffer (PLB), the levels of NF-κB, AP1, and Renilla luciferase activity were detected with the Dual-Luciferase^®^ Reporter (DLR™) Assay System (Promega Biotech, Madison, WI, USA) according to the protocol of the manufacturer.

### Real-Time Quantitative Reverse Transcription PCR

Total RNA was extracted from RAW264.7 cells and BMDM with RNAiso Plus reagent according to the instructions of the manufacturer (TaKaRa, Japan) and reverse transcribed with PrimeScript™ RT reagent Kit (Perfect Real Time) (TaKaRa, Japan). Reverse transcription products of different samples were amplified using a LightCycler 96 system (Roche, Basel, Switzerland) with TB Green^®^ Premix Ex Taq^™^ (Tli RNaseH Plus) (TaKaRa, Japan) according to the instructions of the manufacturer. The 2^−△△Ct^ method was used to calculate the relative mRNA levels normalized to GAPDH or β-actin. The mouse primer sequences were as follows: PHLAD1—forward primer, 5′-GAAGATGGCCCATTCAAAAGCG-3′, reverse primer, 5′-GAGGAGGCTAACACGCAGG-3′; GAPDH—forward primer, 5′-GGTTGTCTCCTGCGACTTCA-3′, reverse primer, 5′-TGGTCCAGGGTTTCTTACTCC-3′.

### Immunoprecipitation and Western Blot

The protein concentration was tested after cell lysis and centrifugation of the whole-cell lysates. Fifty micrograms of protein samples were used as input, and the rest of them were incubated with primary antibodies at 4°C overnight. Then, 20 μl Protein G PLUS-Agarose beads were added into the supernatant containing the above antigen–antibody complex and incubated at room temperature for 4 h. After the above immune complexes were centrifuged and washed, 20 μl 2× loading buffer was added to each sample. Bound proteins were eluted by boiling at 100°C for 10 min and transferred to nitrocellulose membranes after separation of these proteins by sodium dodecyl sulfate-polyacrylamide gel electrophoresis. After the above membranes were incubated with primary antibodies and second antibodies, respectively, a Tanon 5200 Multi Chemiluminescent Imaging System (Tanon, Shanghai, China) was used to detect the protein bands.

### Immunofluorescence

Cells (5 × 10^5^) were seeded into a 3.5-cm dish with glass coverslips. When the cell density reached 80%, cells were fixed with 4% paraformaldehyde for 30 min and then incubated with blocking buffer for 30 min. Cells were incubated with primary antibodies and secondary antibodies, respectively. After the nuclei were stained with DAPI for 15 min, images were taken using the FluoView™ FV1000 Confocal Laser Scanning Microscope (Olympus, Tokyo, Japan).

### Immunohistochemistry

Mouse lung tissues were fixed with 4% formalin buffer and paraffin-embedded after excising. Lung tissues were cut into 4 μm sections, deparaffinized with xylene, and rehydrated with ethanol. The above sections were boiled in antigenic repair solution using a microwave for 20 min. Part of the sections was stained with H&E, the others were incubated 50 ul diluted primary antibodies at 4°C overnight and 50 ul diluted secondary antibodies at room temperature for 30 min. Finally, diaminobenzidine staining was performed. The above sections were imaged under a Leica DMIL FL fluorescent inverted microscope (Leica, Wetzlar, Germany) after hematoxylin redyeing, alcohol dehydration, and clearing in xylene.

### Establishment of a Mouse Model of Endotoxemia

Eighteen BALB/c mice (weight 20–22 g) were randomly divided into three groups, with six mice per group. Groups 2 and 3, as the experimental groups, were intraperitoneally injected with LPS (8 mg/kg), and they were then sacrificed at 1 or 6 h after injection, respectively. Group 1, as the control group, was also sacrificed at 1 h after intraperitoneal injection of the same amount of phosphate buffer saline (PBS). Serums from the above groups of mice were collected and used to detect the levels of proinflammatory cytokines (TNF-α, IL-6, and IL-1β) using ELISA. Lung tissues from the above groups of mice were removed and used to examine PHALD1 expression using immunohistochemistry (IHC) and Western blot.

### Statistical Analysis

SPSS software (version 16.0; SPSS, Inc., Chicago, IL, USA) was used for statistical analysis. All experiments were repeated at least three times. The data were expressed as mean ± SD and compared with independent-samples *t*-test and one-way ANOVA. *P*-values <0.05 were considered to be statistically significant.

## Results

### Kinetics of LPS-Induced PHLDA1 Expression

**Figure 1:**
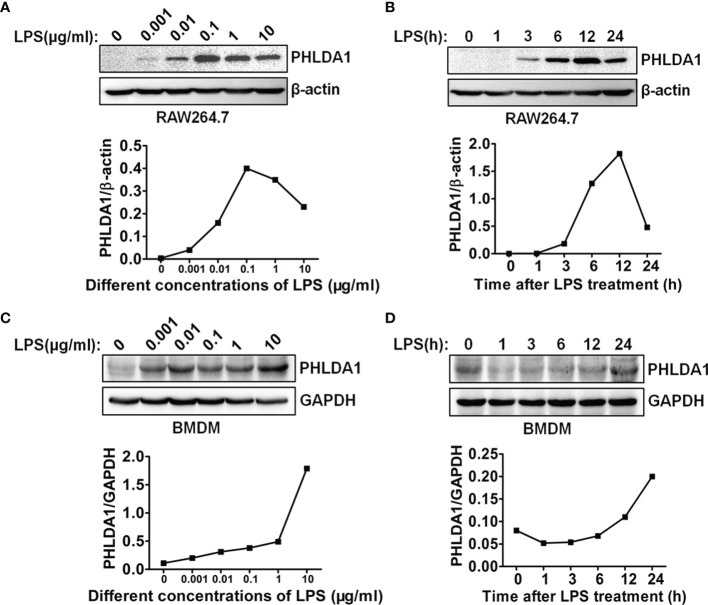
LPS regulates PHLDA1 expression in macrophages. RAW264.7 cells **(A)** and BMDM **(C)** were treated with the different concentrations (0.001, 0.01, 0.1, 1, and 10 μg/ml) of LPS for 24 h. Western blot was used to measure PHLDA1 expression. RAW264.7 cells **(B)** and BMDM **(D)** were treated with LPS (0.1 μg/ml) for 0, 1, 3, 6, 12, and 24 h. Western blot was used to measure PHLDA1 expression. β-Actin or GAPDH was used as a loading control. The data are representative of three independent experiments. The quantified results of PHLDA1 expression are shown in the lower panel.

To investigate the role of PHLDA1 in the response of macrophages to LPS stimulation, we tested whether PHLDA1 expression could be induced by LPS. The results showed that different concentrations of LPS increased significantly PHLDA1 expression in RAW264.7 cells, but two higher concentrations (1 and 10 μg/ml) of LPS did not increase further the expression level of PHLDA1 after PHLDA1 expression reached the peak level with 0.1 μg/ml of LPS treatment for 12 h (**Figure 1A**). Therefore, 0.1 μg/ml was regarded as the concentration at which LPS was used to stimulate RAW264.7 cells. To explore the kinetics of the regulation of PHLDA1 expression in RAW264.7 cells induced by LPS, we tested PHLDA1 expression in RAW264.7 cells treated with LPS for different time periods with Western blot. The results indicated that PHLDA1 expression reached the peak level after 0.1 μg/ml of LPS treatment for 12 h and then gradually decreased (**Figure 1B**). The same experiments were performed in BMDM. The results showed that different concentrations of LPS increased sustainably PHLDA1 expression in BMDM, unlike RAW264.7 cells (**Figure 1C**). In addition, after LPS treatment for different time periods, PHLDA1 expression decreased firstly and then sustainably increased after LPS treatment for 1 h, which was different from that of RAW264.7 cells (**Figure 1D**). In summary, PHLDA1 expression displayed an increasing trend regardless of LPS treatment for different time periods or at different concentrations in both RAW264.7 cells and BMDM. To explore the effect of LPS on proinflammatory cytokine production, we explored LPS concentration- and time-dependent effects on TNF-α production in RAW264.7 cells and BMDM. The results indicated that different concentrations of LPS increased significantly TNF-α production in RAW264.7 cells and BMDM. TNF-α production reached the peak level after 0.1 or 0.01 μg/ml of LPS treatment for 12 h in RAW264.7 cells or BMDM, respectively, and then gradually decreased (**Supplementary Figure 1A**). In addition, LPS also markedly enhanced TNF-α production in RAW264.7 cells and BMDM at different time periods. TNF-α production simultaneously reached the peak level after LPS treatment for 12 h in RAW264.7 cells and BMDM and then gradually decreased (**Supplementary Figure 1B**).

### PHLDA1 Negatively Regulates LPS-Induced Proinflammatory Cytokine Production

**Figure 2:**
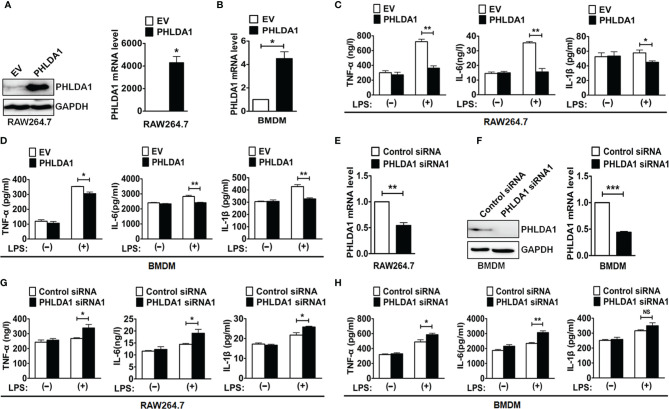
PHLDA1 attenuates LPS-initiated production of proinflammatory cytokines. RAW264.7 cells **(A)** and BMDM **(B)** were transfected with EV or PHLDA1 plasmid. PHLDA1 expression was measured at the protein and mRNA levels with Western blot (left panel) and/or RT-qPCR (right panel). RAW264.7 cells **(C)** and BMDM **(D)**, which were transfected with EV or PHLDA1 plasmid, were treated with or without LPS (0.1 μg/ml) for 12 h. ELISA was performed to measure the production of proinflammatory cytokines (TNF-α, IL-6, and IL-1β). RAW264.7 cells **(E)** and BMDM **(F)** were transfected with Control siRNA and PHLDA1 siRNA1, and PHLDA1 expression was measured at the protein and mRNA levels with Western blot (left panel) and/or RT-qPCR (right panel). RAW264.7 cells **(G)** and BMDM **(H)**, which were transfected with Control siRNA and PHLDA1 siRNA1, were treated with or without LPS (0.1 μg/ml) for 12 h. ELISA was performed to measure the production of proinflammatory cytokines (TNF-α, IL-6, and IL-1β). Data are shown as means ± SD of three independent experiments (NS means no significance, *P < 0.05; **P < 0.01; ***P < 0.001).

To test whether PHLDA1 was involved in the regulation of TLR4 signaling pathway, we overexpressed PHLDA1 using plasmid transfection in RAW264.7 cells and BMDM and then detected the efficiency of PHLDA1 overexpression with Western blot and real-time quantitative reverse transcription PCR (RT-qPCR). The results demonstrated that PHLDA1 expressions were significantly upregulated at both protein (**Figure 2A**, left panel) and mRNA (**Figures 2A**, right panel, and **2B**) levels in the above cells. It should be pointed out that PHLDA1expression was not detected at the protein level using Western blot after PHLDA1 overexpression in BMDM because of limited number of cells. To determine the effect of PHLDA1 overexpression on the production of proinflammatory cytokines including TNF-α, IL-6, and IL-1β, RAW264.7 cells were overexpressed with empty vector (EV) or PHLAD1 plasmid, and then treated with or without LPS for 12 h. The results demonstrated that the production of the proinflammatory cytokines in PHLDA1-overexpressed RAW264.7 cells was significantly decreased after LPS treatment, compared with that of control cells transfected with EV (**Figure 2C**). Similar results were obtained in BMDM after PHLDA1 overexpression (**Figure 2D**). Subsequently, we screened the efficient siRNA fragments for downregulation of PHLAD1 expression. We tested LPS-induced PHLDA1 expression at the protein level with Western blot after transfection of Control siRNA and two PHLDA1 siRNAs (PHLDA1 siRNA1 and siRNA2) because our previous results confirmed that PHLDA1 expression was not detected at the protein level without LPS treatment or in 60 min after LPS treatment. The result demonstrated that both PHLDA1 siRNA1 and PHLDA1 siRNA2 reduced PHLDA1 expression, respectively, compared with Control siRNA in RAW264.7, and the PHLDA1 siRNA1 efficiency of downregulation of PHLDA1 expression was more significant than that of PHLDA1 siRNA2 (**Supplementary Figure 2**). For this reason, we used PHLDA1 siRNA1 to complete the follow-up experiments. To determine the effect of PHLDA1 knockdown on the production of proinflammatory cytokines, we reduced PHLDA1 expression by siRNA interference in RAW264.7 cells and BMDM, and then treated RAW264.7 cells with or without LPS for 12 h. The results demonstrated that the production of proinflammatory cytokines was significantly increased in PHLDA1-deficient RAW264.7 cells after LPS treatment, compared with that of control cells transfected with Control siRNA (**Figures 2E–G**). Similar results were obtained in BMDM after the reduction of PHLDA1 expression (**Figure 2H**).

### PHLDA1 Inhibits the Phosphorylation of Important Signal Molecules in TLR4/MyD88-Mediated Downstream MAPK and NF-κB Signaling Pathways

**Figure 3:**
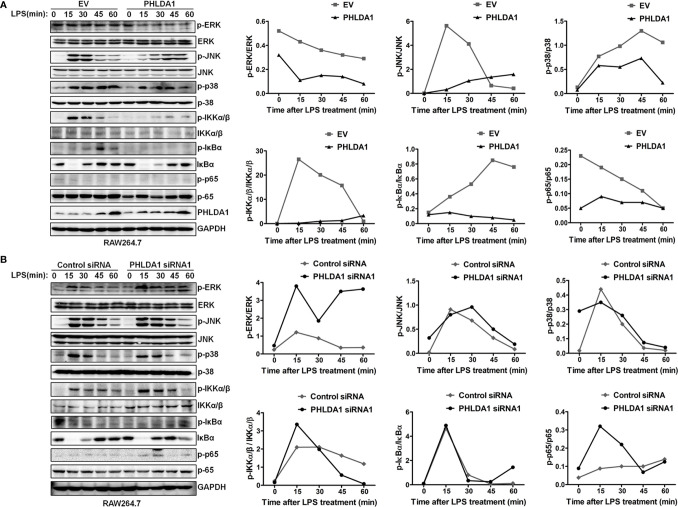
PHLDA1 attenuates the activation of some signal molecules in MyD88-dependent TLR4 signaling pathway. **(A)** RAW264.7 cells were transfected with EV or PHLDA1 plasmid, and then stimulated with LPS (0.1 μg/ml) for the indicated times. Phosphorylation levels and total protein expressions of important signal molecules (ERK, JNK, p38, IKKα/β, IkBa and p65) in cell lysates were analyzed using Western blot. **(B)** RAW264.7 cells were transfected with Control siRNA or PHLDA1 siRNA1, and then stimulated with LPS (0.1 μg/ml) for the indicated times. Phosphorylation levels and total protein expressions of the above molecules were analyzed using Western blot. Data shown are presentative of three independent experiments. Phosphorylation levels of the above molecules were quantitated and shown in the right panel. GAPDH was used as a loading control.

We explored the effect of PHLDA1 on the activation of downstream MAPK and NF-κB signaling pathways. The results showed that PHLDA1 overexpression significantly decreased the phosphorylation levels of some protein molecules (ERK, JNK, p38, IKKα/β, IκBα, and NF-κB subunit p65) in the MAPK and NF‐κB signaling pathways at different time points (0, 15, 30, 45, and 60 min) in RAW264.7 cells after LPS treatment, compared with that of control cells (**Figure 3A**). Then, we further solidified the above findings by including early time point such as 2 and 5 min of LPS stimulation. Similar results were obtained in PHLDA1-upregulated RAW264.7 cells treated with LPS (**Supplementary Figure 3A**). Furthermore, we examined the effect of PHLDA1 knockdown on phosphorylation levels of the above signal molecules, and reverse results were obtained in PHLDA1-deficient RAW264.7 cells treated with LPS (**Figure 3B** and **Supplementary Figure 3B**).

### PHLDA1 Impairs LPS-Induced NF-κB and AP1 Nuclear Translocation and Their Responsive Element Activities

**Figure 4:**
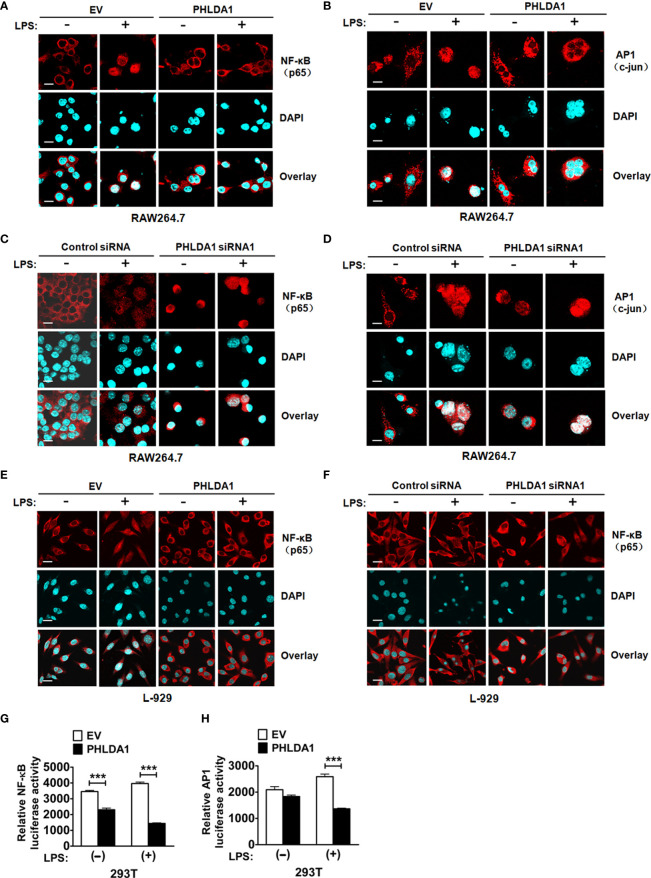
PHLDA1 attenuates LPS-initiated nuclear translocations and responsive element activities of NF-κB and AP1. RAW264.7 cells **(A, B)** and L-929 cells **(E)** were transfected with EV or PHLDA1 plasmid, and then stimulated with or without LPS (0.1 μg/ml) for 1 h. Cells were immunostained with anti-NF-κB (p65) antibody or anti-AP1 (c-jun) antibody and Alexa-594-labeled secondary antibodies. The nuclei were stained with DAPI for 15 min. The merged images were captured with a confocal microscope (scale bar, 20 μm). RAW264.7 cells **(C, D)** and L-929 cells **(F)** were transfected with Control siRNA or PHLDA1 siRNA1, and then stimulated with or without LPS (0.1 μg/ml) for 1 h. Cells were immunostained with anti-NF-κB (p65) antibody or anti-AP1 (c-jun) antibody and Alexa-594-labeled secondary antibodies. The nuclei were stained with DAPI for 15 min. The merged images were captured with a confocal microscope (scale bar, 20 μm). **(G, H)** EV or PHLDA1 plasmid was transfected into 293T cells together with pTK–Renilla luciferase and NF-κB luciferase reporter plasmids. After 24 h of culture, the cells were incubated with LPS (0.1 μg/ml) for 20 h. The Dual-Luciferase^®^ Reporter (DLR™) Assay System was performed to measure NF-κB or AP1 luciferase activity. Data are presented as mean ± SD of three independent experiments (***P < 0.001).

To further explore the underlying mechanism of PHLDA1-affected TLR4/MyD88 signaling pathway, we detected the nuclear translocation of transcription factors NF-κB subunit p65 and AP1 subunit c-jun with immunofluorescence (IF) after upregulation of PHLDA1 expression in RAW264.7 and L-929 cells. The results showed that PHLDA1 overexpression, which was established with IF (**Supplementary Figures 4A, B**), suppressed the translocations of p65 and c-jun from the cytoplasm to nuclear, compared with that of control RAW264.7 and L-929 cells (**Figures 4A, B, E**). The reverse results were obtained in PHLDA1-deficient RAW264.7 and L-929 cells (**Figures 4C, D, F** and **Supplementary Figure 4B**). We then tested the effect of PHLDA1 overexpression on NF-κB and AP1 luciferase activities in 293T cells with or without LPS treatment. The results indicated that PHLDA1 overexpression decreased LPS-induced transcriptional activities of NF-κB and AP1 (**Figures 4G, H**).

### PHLDA1 Inhibits TLR4-Mediated NF-κB Nuclear Translocation and Proinflammatory Cytokine Production Through Binding to Tollip

**Figure 5:**
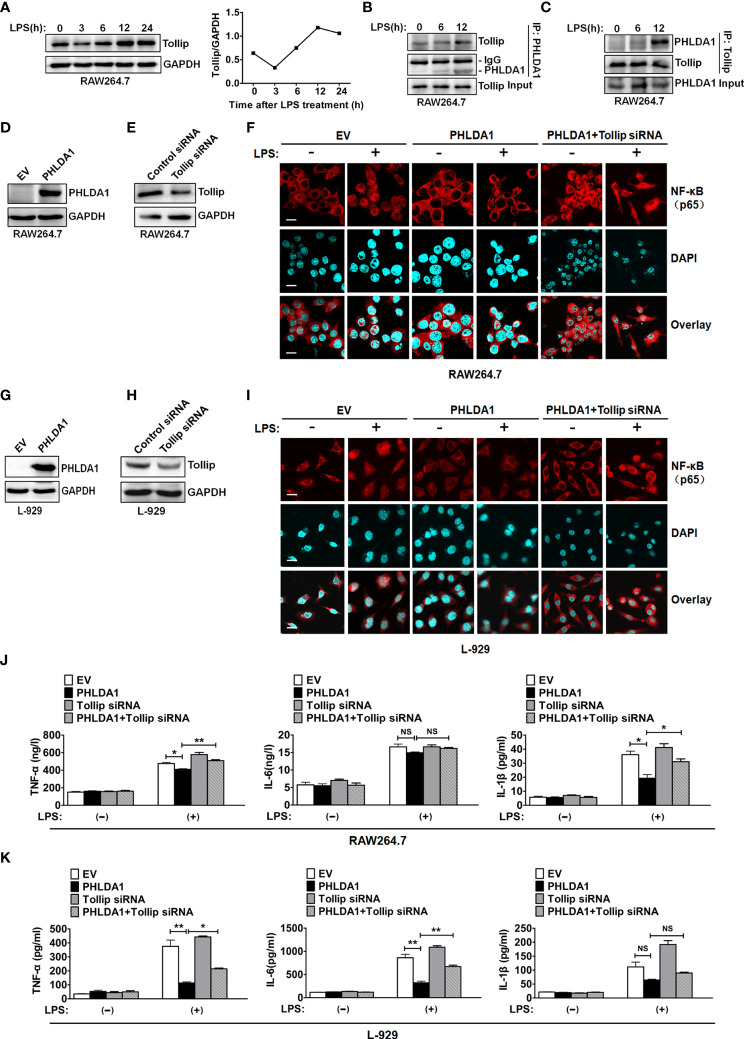
PHLDA1 recruits Tollip to attenuate LPS-initiated NF-κB nuclear translocation and proinflammatory cytokine production. **(A)** RAW264.7 cells were treated with LPS (0.1 μg/ml) for 0, 3, 6, 12, and 24 h. Tollip expression was detected with Western blot. GAPDH was used as loading control. The quantified result of Tollip expression is shown in the right panel. **(B, C)** RAW264.7 cells were treated with 0.1 μg/ml LPS for 0, 6, and 12 h. Co-IP and Western blot analysis were used to measure the interaction between PHLDA1 and Tollip. RAW264.7 cells **(D, E)** and L-929 cells **(G, H)** were transfected with EV or PHLDA1 plasmid, Control siRNA, or Tollip siRNA, respectively. Protein expressions of PHLDA1 and Tollip were measured with Western blot. GAPDH was used as an internal control for gene expression analysis. RAW264.7 cells **(F)** and L-929 cells **(I)** were transfected with EV, PHLDA1 plasmid, and PHLDA1 plasmid plus Tollip siRNA and then treated with LPS (0.1 μg/ml) for 1 h. The above cells were fixed and stained for NF-κB (p65). Nuclei were stained with DAPI. The merged images were captured with a confocal microscope (scale bar, 20 μm). RAW264.7 cells **(J)** and L-929 cells **(K)** were transfected with EV, PHLDA1 plasmid, Tollip siRNA, and PHLDA1 plasmid plus Tollip siRNA and then treated with LPS (0.1 μg/ml) for 12 h. ELISA was performed to measure the production of proinflammatory cytokines (TNF-α, IL-6, and IL-1β). Data are shown as mean ± SD of three independent experiments (NS means no significance, *P < 0.05; **P < 0.01). Western blot data are representative of three independent experiments.

Tollip, an important negative regulator of innate immunity, suppresses TLR signaling pathway through impairing the activity of interleukin-1 receptor-associated kinase (IRAK) (26–28). To investigate the role of Tollip which played in PHLDA1-impaired proinflammatory cytokine production induced by LPS, we completed the following *in-vitro* experiments. We found that Tollip expression presented an increasing trend as a whole in RAW264.7 treated with LPS for different time periods (**Figure 5A**). Co-IP assay was used to examine the effect of LPS treatment on the interaction between PHLDA1 and Tollip. The results showed that the above interaction gradually strengthened along with the increase of LPS treatment time (**Figures 5B, C**). Furthermore, we tested NF-κB nuclear translocation induced by LPS based on PHLDA1 overexpression and Tollip downregulation. IF results confirmed that PHLDA1 overexpression restrained LPS-induced NF-κB translocation from the cytoplasm to nuclear, compared with that of control RAW264.7 and L-929 cells. On the contrary, knockdown of Tollip reversed the above biological phenomenon (**Figures 5D–F**). The same experiments were performed with L-929 cells and similar results were obtained (**Figures 5G–I**). Then, we tested the production of proinflammatory cytokines (TNF-α, IL-6, and IL-1β) induced by LPS based on PHLDA1 overexpression and Tollip downregulation. The results demonstrated that PHLDA1 overexpression restrained LPS-induced proinflammatory cytokine production, while knockdown of Tollip reversed the above biological phenomenon (**Figure 5J**). The same experiments were performed with L-929 cells and similar results were obtained (**Figure 5K**).

### Verification of the Anti-Inflammatory Effect of PHLDA1 *In Vivo*

**Figure 6:**
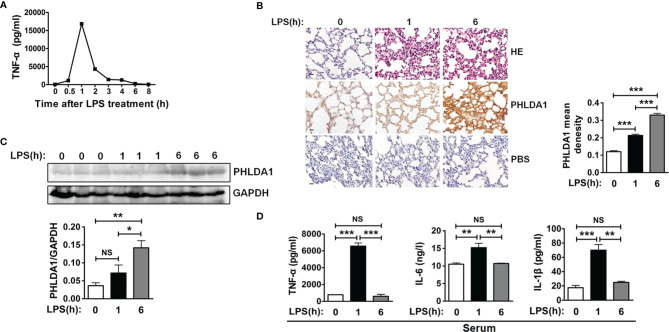
PHLDA1 alleviates LPS-initiated proinflammatory cytokine production in a mouse model of endotoxemia. **(A)** ELISA was performed to measure TNF-α level in serum from mice treated with LPS (8 mg/kg) for 0, 0.5, 1, 2, 3, 4, 6, and 8 h, respectively. **(B)** H&E and PHLDA1 staining (scale bar, 20 μm) of lung tissues from three groups of mice, including group 1 (control group), group 2 (treatment with LPS for 1 h), and group 3 (treatment with LPS for 6 h). PHLDA1 mean density was analyzed and shown in the right panel. **(C)** Western bolt analysis of PHLDA1 in the lung tissues from the above different groups of mice. The quantified result of PHLDA1 expression is shown in the lower panel. **(D)** ELISA analysis of proinflammatory cytokines (TNF-α, IL-6, and IL-1β) in serum from the above different groups of mice. The data, including quantitative analysis of PHLDA1 expression, PHLDA1 mean density, and ELISA, are expressed as mean ± SD from three independent experiments. (NS means no significance, *P < 0.05; **P < 0.01; ***P < 0.001).

We used an endotoxemia mouse model to further confirm the above action mechanism of PHLDA1 in innate immunity after a series of *in-vitro* experiments. First of all, we screened the optimum LPS-treated time for constructing the above mouse model by detecting the kinetics of TNF-α concentration in serum from mice treated by LPS for different time periods. The results demonstrated that TNF-α concentration increased originally and reached the peak level after LPS treatment for 1 h, and then gradually decreased. TNF-α concentration is close to zero (**Figure 6A**). So 1 and 6 h were used as LPS treatment time to construct the above mouse model. We then examined PHLDA1 expressions of lung tissues from different groups of mice injected by LPS. IHC results showed that PHLDA1 expressions of lung tissues from group 2 (mice treated with LPS for 1 h) were more than those from group 1 (mice treated with PBS for 1 h), and PHLDA1 expressions of lung tissues from group 3 (mice treated with LPS for 6 h) were more than those from group 2 (**Figure 6B**). Similar results were obtained in the lung tissues from mice by Western blot analysis (**Figure 6C**). ELISA analysis indicated that the levels of proinflammatory cytokines (TNF-α, IL-6, and IL-1β) of serum from group 2 were higher than those from group 1, while the levels of proinflammatory cytokines from group 3 were lower than those from group 2 (**Figure 6D**).

## Discussion

**Figure 7:**
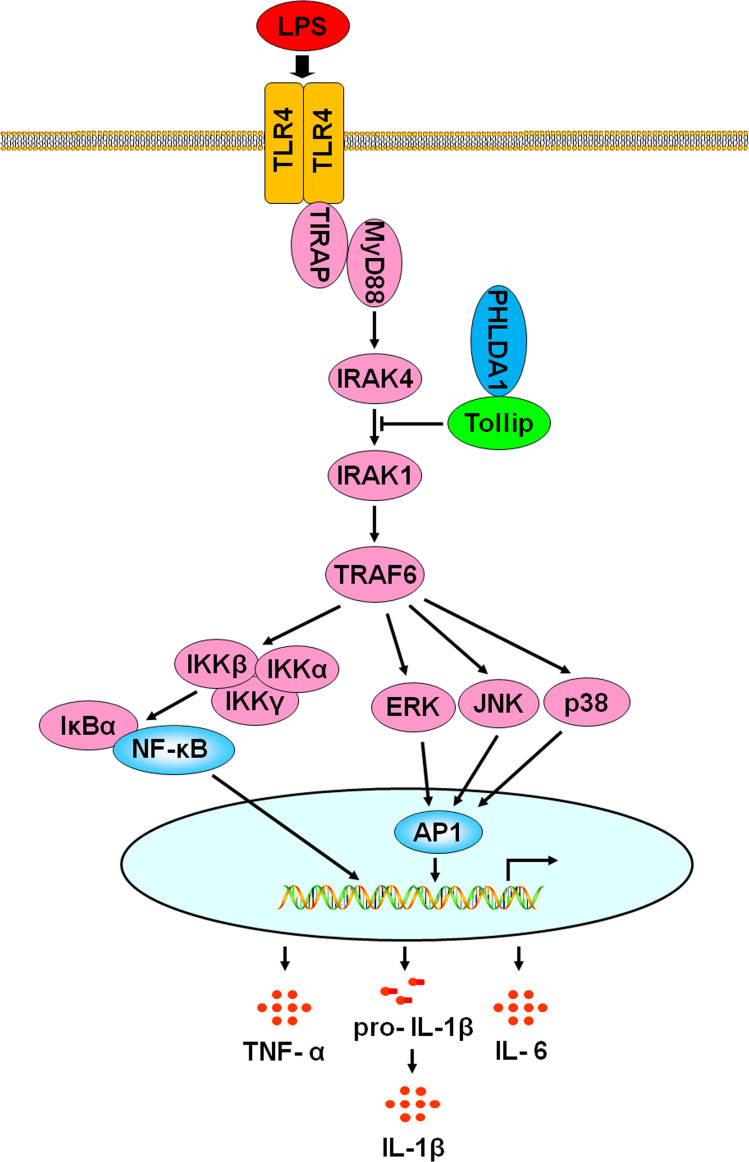
A schematic representation of the role of PHLDA1 in modulating TLR4-mediated proinflammatory cytokine production *via* Tollip. After LPS-induced TLR4 activation, PHLDA1 recruited Tollip to inhibit IRAK phosphorylation, which further decreased the phosphorylation levels of some signal molecules (ERK, JNK, p38, IKKα/β, IκBα, and NF-κB subunit p65), the abilities of NF-κB and AP1 nuclear translocations, and their responsive element activities. Eventually, LPS-initiated proinflammatory cytokine production was repressed.

In this study, we found that PHLDA1 expression was low and showed time- and dose-dependent changes after being exposed to LPS, suggesting that it was potentially involved in the regulation of TLR4 signaling pathway. Further studies demonstrated that overexpression of PHLDA1 attenuated LPS-induced proinflammatory cytokine production and vice versa. PHLDA1 impaired the phosphorylation levels of some important molecules (ERK, JNK, p38, IKKα/β, IκBα, and NF-κB subunit p65) in TLR4/MyD88-dependent signaling pathway, which in turn restrained the translocations of NF-κB and AP1 from the cytoplasm to nuclear and their responsive element activities. Finally, we found that PHLDA1 recruited Tollip to restrain LPS-induced proinflammatory cytokine production. Similar results were obtained *in vivo*. These findings demonstrate that PHLDA1 has a pivotal role in regulating LPS-induced proinflammatory cytokine production. The proposed mechanism is depicted in **Figure 7**.

Previous studies have explored the role of PHLDA1 in inflammation. PHLDA1 was reported to positively regulate TLR2 signaling pathway to enhance lung contusion (29). Han et al. found that PHLDA1 directly interacted with TRAF6 and augmented K63-linked ubiquitination to activate the NF-κB signaling pathway, which in turn promoted the production of LPS-induced proinflammatory cytokines (TNF-α and IL-1β) and associated genes (iNOS and COX-2) in microglia cells. These findings indicated that PHLDA1 may be a potent regulator for neuroinflammation (30, 31). It was reported that PHLDA1 upregulation was dependent of RAW264.7 cell proliferation and cell cycle progression induced by LPS (32). Pam3CSK4, as a TLR2 ligand, induced PHLDA1 expression which was modulated *via* the JAK2–ERK1/2–STAT3 signaling pathway (33). Hossain et al. reported that PHLDA1 knockout significantly impaired LPS-induced expression of MCP-1 but not TNF-α (9), which is different from our results. In our study, the results showed that PHLDA1 knockdown enhanced LPS-induced production of the proinflammatory cytokines (TNF-α, IL-6, and IL-1β). The possible reasons are as follows: 1) cells used in the experiments were different. RAW264.7 cell lines and BMDM isolated from C57BL/6J mice were used in our study, while peritoneal macrophages isolated from C57BL/6J mice were used in the report mentioned above; 2) gene regulation was different. RNA interference technology was used to downregulate PHLDA1 expression in our study, while gene knockout technology was used to delete PHLDA1 gene in the report mentioned above.

Tollip, one of the important adaptor molecules, contains three different domains including Tom1-binding domain, conserved 2 domain, and coupling of ubiquitin to ER degradation domain (34). In human, Tollip has four isoforms, namely, isoform A, isoform B, isoform C, and isoform D (35). Tollip plays a critical role in regulating TLR-mediated innate immune responses (36, 37). It was reported that Tollip interacted directly with TLR2 or TLR4 with its C-terminal domain (179–273 aa). Tollip impaired TLR-mediated cell activation and the production of proinflammatory cytokines through suppressing phosphorylation and kinase activity of IRAK in inflammation. In addition, the C-terminal region of Tollip was phosphorylated by IRAK after LPS stimulation (26, 38). Liu et al. found that lysine histone methyltransferase EZh1 enhanced the production of inflammatory cytokines (IL-6, TNF-α, and IFN-β) by inhibiting Tollip (39). Tollip knockout increased the ability of clear *Legionella pneumophila* (Lp) and proinflammatory cytokine production by affecting TLR2 activity in Lp-infected mice (40). Monophosphoryl lipid A inhibited the LPS-triggered signaling pathway *via* the PI3K-dependent induction of Tollip, which in turn reduced the phosphorylation and activation of IRAK-1 to impair TLR4–MyD88 signaling pathway (41–43). The above results suggested that Tollip inhibited TLR2 and TLR4 signaling pathways. In this study, we established that PHLDA1 negatively regulated the TLR4/MyD88-dependent signaling pathway. Then, we explored the relationship between Tollip and PHLDA1. We found that PHLDA1 interacted with Tollip, and the LPS-induced interaction between the above molecules gradually increased in a time-dependent manner. To investigate whether PHLDA1 suppressed TLR4/MyD88-dependent signaling pathway through Tollip, we detected LPS-induced production of proinflammatory cytokines after PHLDA1 overexpression and Tollip downregulation, and the results showed that PHLDA1 overexpression reduced LPS-induced proinflammatory cytokine production, while Tollip deficiency rescued the above biological phenomenon. These findings confirmed our hypothesis.

In summary, PHLDA1 recruited Tollip to negatively modulate the phosphorylation levels of some important molecules (ERK, JNK, p38, IKKα/β, IκBα, and NF-κB subunit p65) in the TLR4/MyD88 signaling pathway, which in turn suppressed NF-κB and AP1 nuclear translocation and their responsive element activities. Finally, LPS-induced proinflammatory cytokine production was diminished. Our study provides a new vision for exploring the function of PHLDA1 and a new therapeutic target for endotoxemia. Further studies should be conducted to investigate the following aspects: 1) the effect of PHLDA1 on TLR4, Tollip, and adaptor proteins (TIRAP, MyD88, IRAK1, IRAK4, and TRAF6); 2) the role of PHLDA1 in downstream TRIF-dependent signaling pathway; 3) the effect of PHLDA1 on the phagocytosis of macrophage; and 4) the discovery and development of effective adenovirus-mediated PHLDA1 injection.

## Data Availability Statement

The original contributions presented in the study are included in the article/**Supplementary Material**. Further inquiries can be directed to the corresponding authors.

## Ethics Statement

The animal study was reviewed and approved by the Ethics Committee of Youjiang Medical University for Nationalities.

## Author Contributions

JW conceived the research and designed the experiments. HP, JH, HH, FW, ZX, JM, JG, JZ and NN performed the experiments. HP wrote the manuscript. BL, XS and DH analyzed the data. All authors revised the manuscript and approved its final version.

## Funding

This work was supported by grants of the National Natural Science Foundation of China (81760513 and 82060528), the Guangxi Natural Science Foundation of China (2019JJA140532), the National and Provincial Natural Science Foundation Cultivation Special Project (210719166884337 and 210719166884340), Li Ka-Shing Foundation Cross-Disciplinary Research Grant (2020LKSFG09B).

## Conflict of Interest

The authors declare that the research was conducted in the absence of any commercial or financial relationships that could be construed as a potential conflict of interest.

## Publisher’s Note

All claims expressed in this article are solely those of the authors and do not necessarily represent those of their affiliated organizations, or those of the publisher, the editors and the reviewers. Any product that may be evaluated in this article, or claim that may be made by its manufacturer, is not guaranteed or endorsed by the publisher.

## Supplementary Material

Supplementary Figure 1 Kinetics of TNF-α level in the culture supernatants of LPS-stimulated RAW264.7 cells and BMDM. **(A)** RAW264.7 cells and BMDM were treated with various concentrations of LPS (0, 0.001, 0.01, 0.1, 1 and 10 µg/ml) for 12 h. TNF-α in cell culture supernatants was detected using ELISA. **(B)** RAW264.7 cells and BMDM were treated with LPS (0.1 µg/ml) for 0, 1, 3, 6, 12 and 24 h. TNF-α in cell culture supernatants was detected using ELISA.

Supplementary Figure 2 Screening of PHLDA1 siRNA fragments RAW264.7 cells were transfected with Control siRNA or 2 PHLDA1 siRNA fragments (PHLDA1 siRNA1, 2) and stimulated with LPS (0.1 µg/ml) for 12 h. PHLDA1 protein expression was detected with Western blot. The quantified result of PHLDA1 expression was shown in the right panel.

Supplementary Figure 3 The effect of PHLDA1 on phosphorylation level of p65 and analysis of PHLDA1mRNA expression after RNA interference. **(A)** RAW264.7 cells were transfected with EV or PHLDA1 plasmid stimulated with LPS (0.1 µg/ml) for the indicated times. Phosphorylation level of p65 and protein expressions of PHLDA1 and p65 in cell lysates were detected with Western blot. GAPDH was used as loading control. **(B)** RAW264.7 cells were transfected with Control siRNA or PHLDA1 siRNA and stimulated with LPS (0.1 μg/ml) for the indicated times. PHLDA1mRNA expression was detected with RT-qPCR.

Supplementary Figure 4 IF analysis of PHLDA1 overexpression and silencing **(A)** RAW264.7 cells were transfected with EV or PHLDA1 plasmid. The above cells were fixed and stained for PHLDA1. Nuclei were stained with DAPI. The merged images were viewed with a confocal microscope (Scale bar, 20 μm). **(B)** L-929 cells were transfected with EV, PHLDA1 plasmid, Control siRNA and PHLDA1 siRNA, respectively. The above cells were fixed and stained for PHLDA1. Nuclei were stained with DAPI. The merged images were viewed with a confocal microscope (Scale bar, 20 μm).
