# Corrigendum: PHLDA1 Suppresses TLR4-Triggered Proinflammatory Cytokine Production by Interaction With Tollip

**DOI:** 10.3389/fimmu.2022.877352

**Published:** 2022-03-15

**Authors:** Hui Peng, Juping Wang, Xuhong Song, Jiangni Huang, Haoming Hua, Fanlu Wang, Ziyun Xu, Jing Ma, Jie Gao, Jing Zhao, Anna Nong, Dongyang Huang, Bin Liang

**Affiliations:** ^1^ Department of Cell Biology and Genetics, Key Laboratory of Molecular Biology in High Cancer Incidence Coastal Chao Shan Area of Guang Dong Higher Education Institutes, Shantou University Medical College, Shantou, China; ^2^ Department of Clinical Laboratory, Affiliated Hospital of Youjiang Medical University for Nationalities, Baise, China; ^3^ Department of Pathophysiology, School of Basic Medical Sciences, Youjiang Medical University for Nationalities, Baise, China

**Keywords:** PHLDA1, TLR4, suppress, proinflammatory cytokine, Tollip

In the original article, there was a mistake in **Figure 3** as published. **“ERK and JNK” was wrongly written as “ERK1/2 and JNK1/2”**. The corrected **Figure 3** appears below.

**Figure 3:**
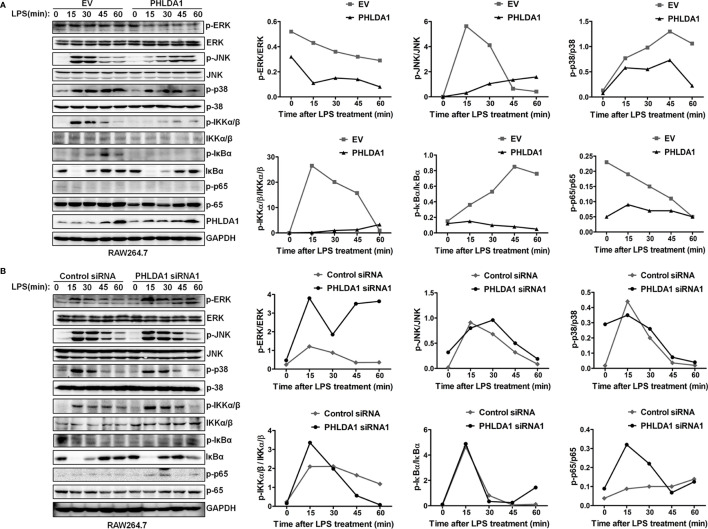
PHLDA1 attenuates the activation of some signal molecules in MyD88-dependent TLR4 signaling pathway. **(A)** RAW264.7 cells were transfected with EV or PHLDA1 plasmid, and then stimulated with LPS (0.1 μg/ml) for the indicated times. Phosphorylation levels and total protein expressions of important signal molecules (ERK, JNK, p38, IKKα/β, IkBa and p65) in cell lysates were analyzed using Western blot. ** (B) ** RAW264.7 cells were transfected with Control siRNA or PHLDA1 siRNA1, and then stimulated with LPS (0.1 μg/ml) for the indicated times. Phosphorylation levels and total protein expressions of the above molecules were analyzed using Western blot. Data shown are presentative of three independent experiments. Phosphorylation levels of the above molecules were quantitated and shown in the right panel. GAPDH was used as a loading control.

In the original article, there was a mistake in **Figure 4** as published. **Figures 4B, F** **were unintentionally flipped over**. The corrected **Figure 4** appears below.

**Figure 4:**
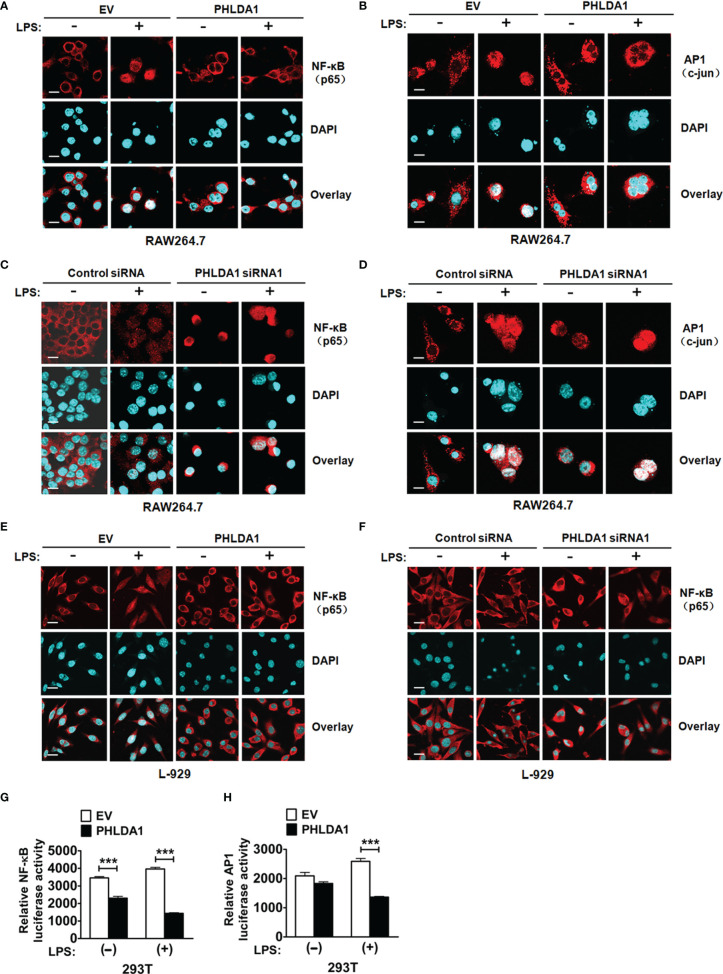
PHLDA1 attenuates LPS-initiated nuclear translocations and responsive element activities of NF-κB and AP1. RAW264.7 cells **(A, B)** and L-929 cells **(E)** were transfected with EV or PHLDA1 plasmid, and then stimulated with or without LPS (0.1 μg/ml) for 1 h. Cells were immunostained with anti-NF-κB (p65) antibody or anti-AP1 (c-jun) antibody and Alexa-594-labeled secondary antibodies. The nuclei were stained with DAPI for 15 min. The merged images were captured with a confocal microscope (scale bar, 20 μm). RAW264.7 cells **(C, D)** and L-929 cells ** (F) ** were transfected with Control siRNA or PHLDA1 siRNA1, and then stimulated with or without LPS (0.1 μg/ml) for 1 h. Cells were immunostained with anti-NF- B (p65) antibody or anti-AP1 (c-jun) antibody and Alexa-594-labeled secondary antibodies. The nuclei were stained with DAPI for 15 min. The merged images were captured with a confocal microscope (scale bar, 20 μm). ** (G, H) ** EV or PHLDA1 plasmid was transfected into 293T cells together with pTK–Renilla luciferase and NF-κB luciferase reporter plasmids. After 24 h of culture, the cells were incubated with LPS (0.1 μg/ml) for 20 h. The Dual-Luciferase® Reporter (DLR™) Assay System was performed to measure NF-κB or AP1 luciferase activity. Data are presented as mean ± SD of three independent experiments (***P < 0.001).

The authors apologize for these errors and state that they do not change the scientific conclusions of the article in any way. The original article has been updated.

## Publisher’s Note

All claims expressed in this article are solely those of the authors and do not necessarily represent those of their affiliated organizations, or those of the publisher, the editors and the reviewers. Any product that may be evaluated in this article, or claim that may be made by its manufacturer, is not guaranteed or endorsed by the publisher.

