# Supplementary figures and images for: PHLDA1 Suppresses TLR4-Triggered Proinflammatory Cytokine Production by Interaction With Tollip
Source: Front Immunol. 2022 Feb 14;13:731500. doi: 10.3389/fimmu.2022.731500 (PMC8882599; doi:10.3389/fimmu.2022.731500)

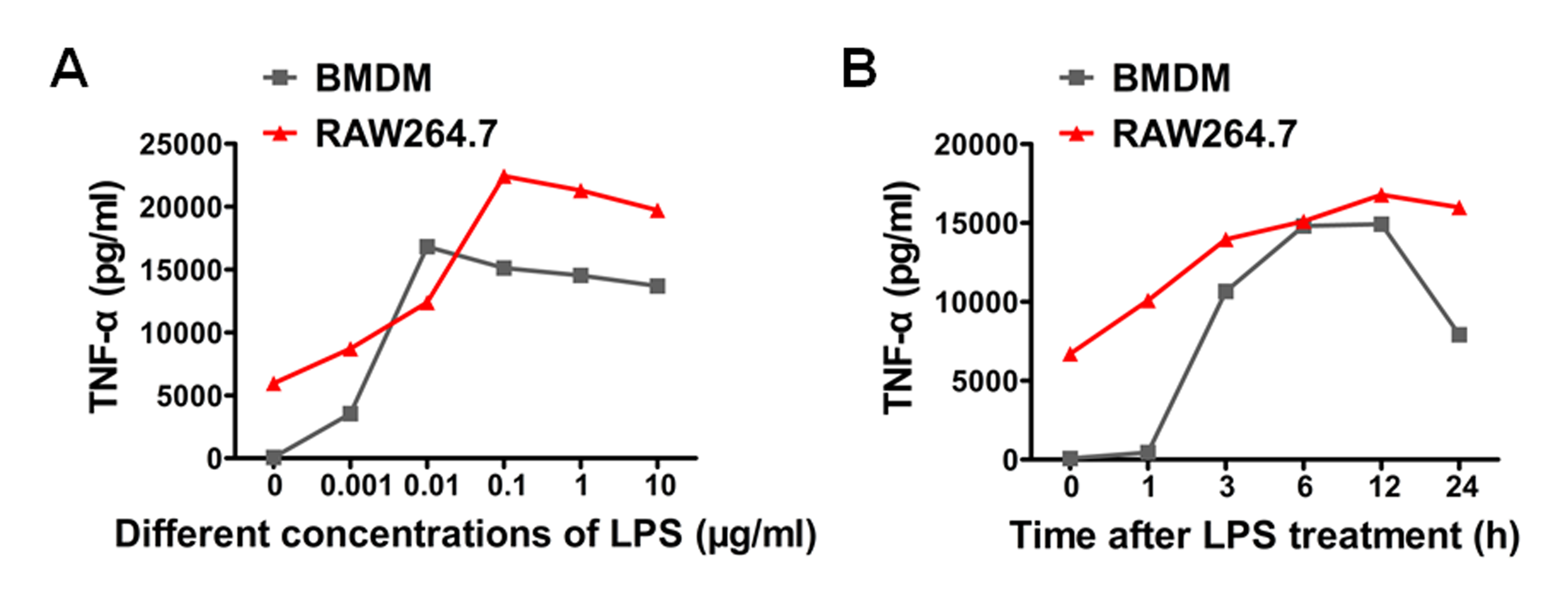

Supplement: Supplementary Figure 1 — Kinetics of TNF-α level in the culture supernatants of LPS-stimulated RAW264.7 cells and BMDM. (A) RAW264.7 cells and BMDM were treated with various concentrations of LPS (0, 0.001, 0.01, 0.1, 1 and 10 µg/ml) for 12 h. TNF-α in cell culture supernatants was detected using ELISA. (B) RAW264.7 cells and BMDM were treated with LPS (0.1 µg/ml) for 0, 1, 3, 6, 12 and 24 h. TNF-α in cell culture supernatants was detected using ELISA. [file Image_1.tif]

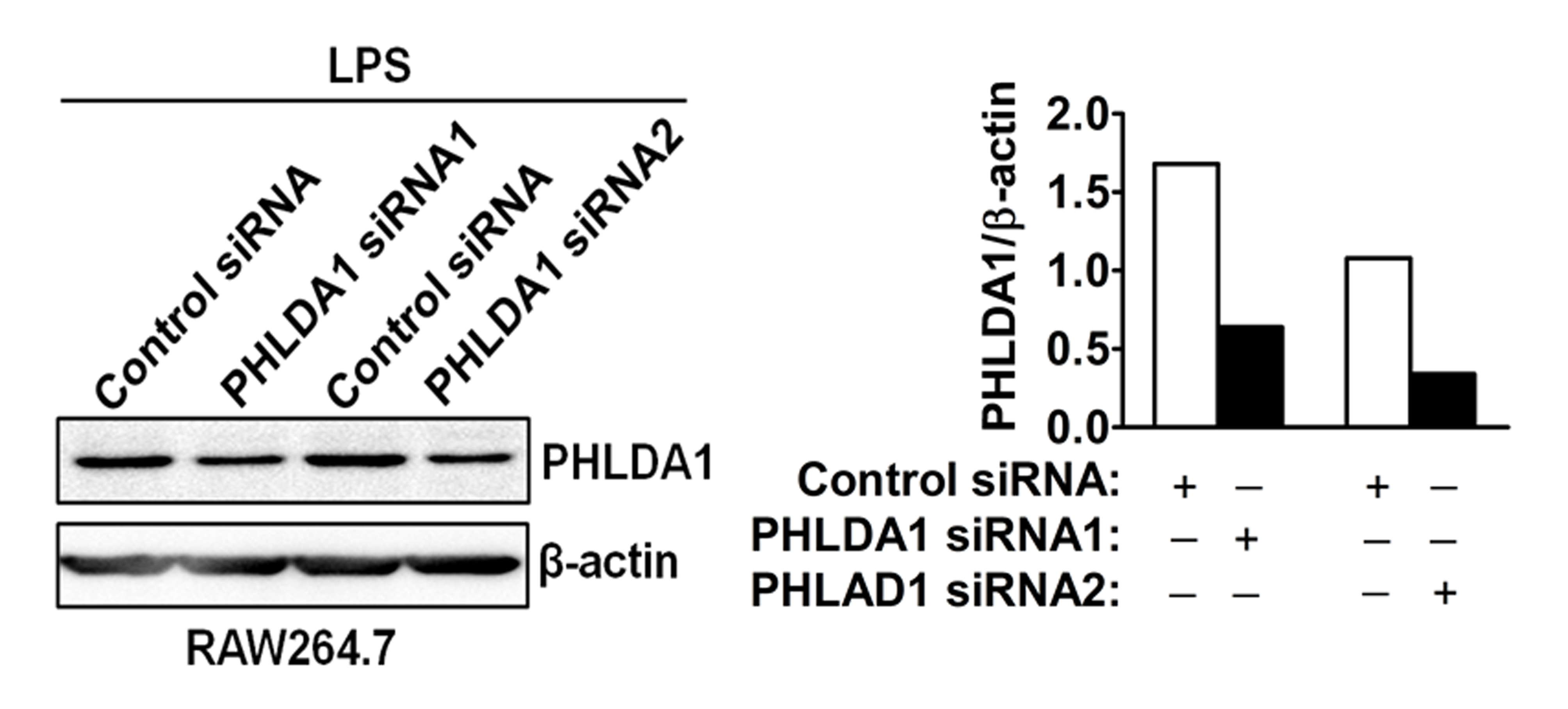

Supplement: Supplementary Figure 2 — Screening of PHLDA1 siRNA fragments RAW264.7 cells were transfected with Control siRNA or 2 PHLDA1 siRNA fragments (PHLDA1 siRNA1, 2) and stimulated with LPS (0.1 µg/ml) for 12 h. PHLDA1 protein expression was detected with Western blot. The quantified result of PHLDA1 expression was shown in the right panel. [file Image_2.tif]

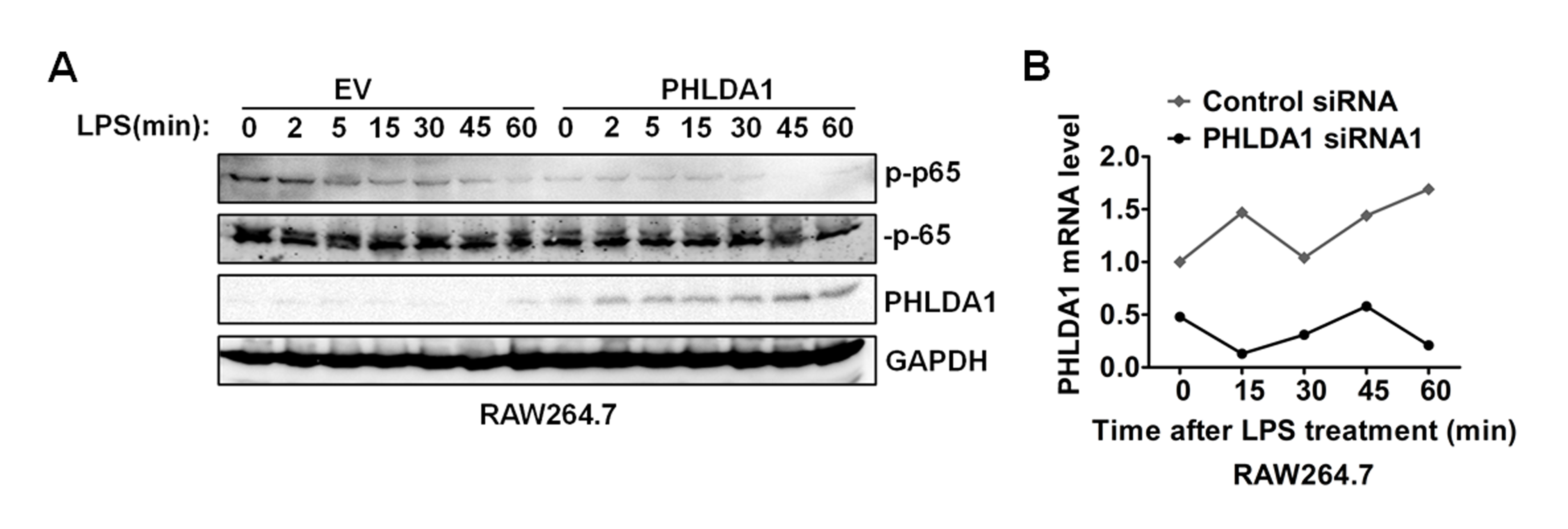

Supplement: Supplementary Figure 3 — The effect of PHLDA1 on phosphorylation level of p65 and analysis of PHLDA1mRNA expression after RNA interference. (A) RAW264.7 cells were transfected with EV or PHLDA1 plasmid stimulated with LPS (0.1 µg/ml) for the indicated times. Phosphorylation level of p65 and protein expressions of PHLDA1 and p65 in cell lysates were detected with Western blot. GAPDH was used as loading control. (B) RAW264.7 cells were transfected with Control siRNA or PHLDA1 siRNA and stimulated with LPS (0.1 μg/ml) for the indicated times. PHLDA1mRNA expression was detected with RT-qPCR. [file Image_3.tif]

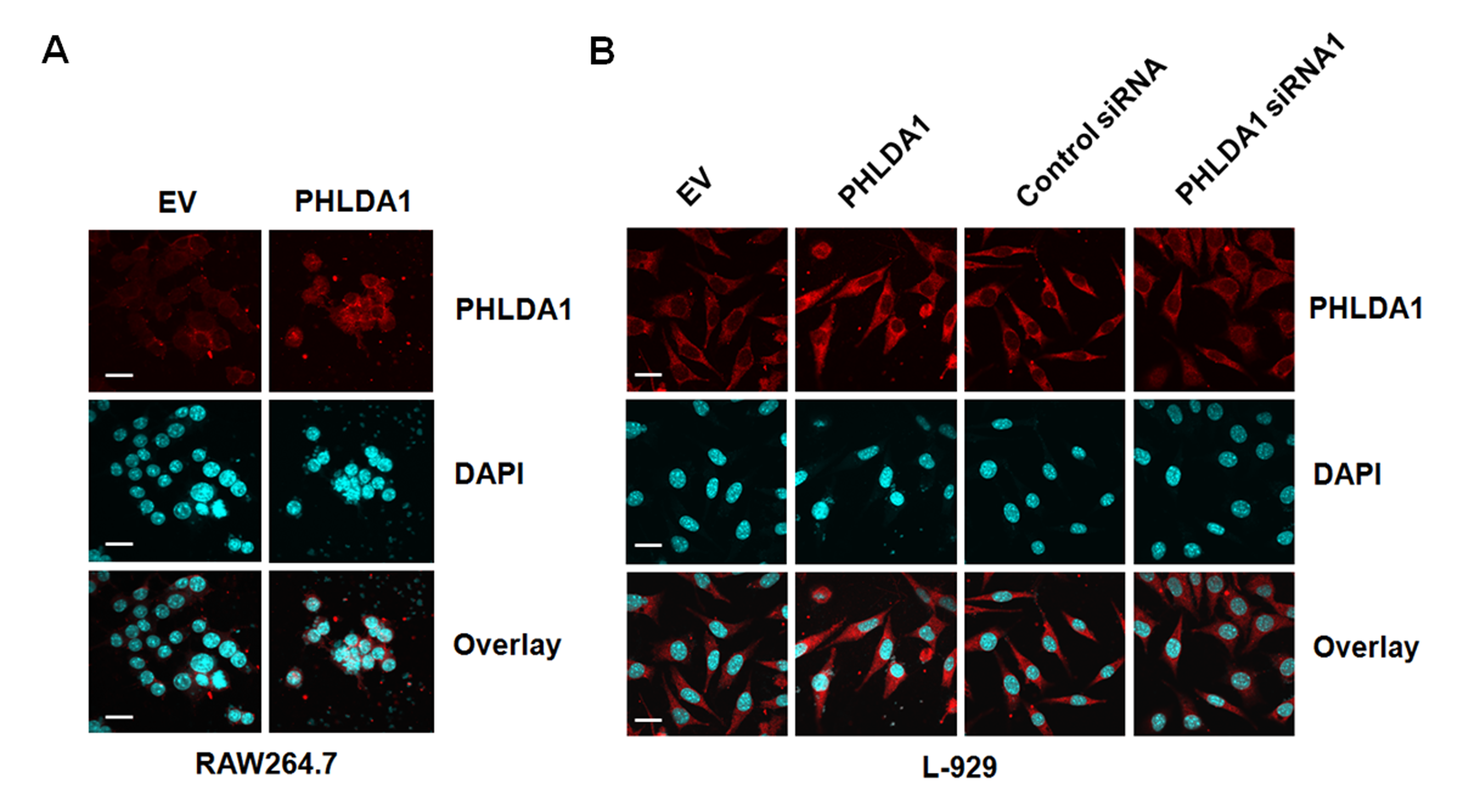

Supplement: Supplementary Figure 4 — IF analysis of PHLDA1 overexpression and silencing (A) RAW264.7 cells were transfected with EV or PHLDA1 plasmid. The above cells were fixed and stained for PHLDA1. Nuclei were stained with DAPI. The merged images were viewed with a confocal microscope (Scale bar, 20 μm). (B) L-929 cells were transfected with EV, PHLDA1 plasmid, Control siRNA and PHLDA1 siRNA, respectively. The above cells were fixed and stained for PHLDA1. Nuclei were stained with DAPI. The merged images were viewed with a confocal microscope (Scale bar, 20 μm). [file Image_4.tif]
