# Supplementary material for: PHLDA1 Suppresses TLR4-Triggered Proinflammatory Cytokine Production by Interaction With Tollip
Source: Front Immunol. 2022 Feb 14;13:731500. doi: 10.3389/fimmu.2022.731500 (PMC8882599; doi:10.3389/fimmu.2022.731500)
Supplement: Supplementary file 5 [file Table_1.doc]

**Supplementary Table 1** **Details of Primary and Second Antibodies**

| **Primary antibody** | **Source** | **Dilution** | **Incubation** | **Supplier** |
| --- | --- | --- | --- | --- |
| PHLDA1 | Mouse | 1:1000 | 4℃, overnight | Santa Cruz Biotechnology, USA |
| Tollip | Rabbit | 1:1000 | 4℃, overnight | ABclonal Technology, China |
| p44/42 MAPK (Erk1/2) | Mouse | 1:1000 | 4℃, overnight | Cell Signaling Technology, USA |
| SAPK/JNK  p38 MAPK  IKKα/β  IκBα  NF-κB p65  Phospho-p44/42 MAPK (Erk1/2) (Thr202/Tyr204) Phospho-SAPK/JNK (Thr183/Tyr185)  Phospho-p38 MAPK (Thr180/Tyr182)  Phospho-IKKα/β (Ser176/180)  Phospho-IκBα (Ser32/36)  Phospho-NF-kB p65/RelA-S536 | Rabbit  Rabbit  Rabbit  Mouse  Rabbit  Mouse  Mouse  Rabbit  Rabbit  Mouse  Rabbit | 1:1000  1:1000  1:1000  1:1000  1:1000  1:1000  1:1000  1:1000  1:1000  1:1000  1:1000 | 4℃, overnight  4℃, overnight  4℃, overnight  4℃, overnight  4℃, overnight  4℃, overnight  4℃, overnight  4℃, overnight  4℃, overnight  4℃, overnight  4℃, overnight | Cell Signaling Technology, USA  Cell Signaling Technology, USA  Cell Signaling Technology, USA  Cell Signaling Technology, USA  Cell Signaling Technology, USA  Cell Signaling Technology, USA  Cell Signaling Technology, USA  Cell Signaling Technology, USA  Cell Signaling Technology, USA  Cell Signaling Technology, USA  ABclonal Technology, China |
| β-actin | Mouse | 1:1000 | 4℃, overnight | Beyotime Biotechnology, China |
| GAPDH | Rabbit | 1:10000 | 4℃, overnight | Proteintech Group, Inc, USA |
| **Second antibody** |  |  |  |  |
| Alexa Fluor® 594-Conjugated  AffiniPure Goat Anti-Rabbit IgG (H+L) |  | 1:300  1:10000 | Room temperature，2h  Room temperature，1h | ZSGB-BIO, China  Proteintech Group, Inc, USA |
| AffiniPure Goat Anti-Mouse IgG (H+L) |  | 1:10000 | Room temperature，1h | Proteintech Group, Inc, USA |
| IRDye 800 CW goat anti-mouse |  | 1:10000 | Room temperature，1h | LI-COR Biosciences, USA |
| IRDye 680 RD goat anti-rabbit |  | 1:10000 | Room temperature，1h | LI-COR Biosciences, USA |
