# Supplementary material for: PHLDA1 Suppresses TLR4-Triggered Proinflammatory Cytokine Production by Interaction With Tollip
Source: Front Immunol. 2022 Feb 14;13:731500. doi: 10.3389/fimmu.2022.731500 (PMC8882599; doi:10.3389/fimmu.2022.731500)
Supplement: Supplementary file 6 [file Table_2.doc]

**Supplementary Table 2** PHLDA1 and Tollip siRNA sequences.

| **siRNA name** | **siRNA sequence 5’ -3’** | **Size(bp)** |
| --- | --- | --- |
| PHLDA1 siRNA1 | Sense 5’ GGUGCAGUACAAGAAUCGUTT 3’ | 21 |
|  | Antisense 5’ ACGAUUCUUGUACUGCACCTT 3’ | 21 |
| PHLDA1 siRNA2 | Sense 5’ GGAGCGAUGAUGUACUGUATT3’ | 21 |
|  | Antisense 5’ UACAGUACAUCAUCGCUCCTT3’ | 21 |
| Tollip siRNA | Sense 5’ CAGGGAAGUAAUCCGUUCUTT 3’ | 21 |
|  | Antisense 5’ AGAACGGAUUACUUCCCUGTT 3’ | 21 |
| Control siRNA | Sense 5’ UUCUCCGAACGUGUCACGUTT 3’ | 21 |
|  | Antisense 5’ACGUGACACGUUCGGAGAATT 3’ | 21 |
